# Distinct profiles of childhood maltreatment and recent stress in relation to non-suicidal self-injury: the roles of self-criticism and self-compassion

**DOI:** 10.3389/fpsyt.2026.1898914

**Published:** 2026-08-26

**Authors:** Min Zong, Helin Zou, Ziyi Yan, Wen Gu, Yi Feng, Zhihong Qiao

**Affiliations:** 1 Mental Health Center, China Foreign Affairs University, Beijing, China; 2 Paula and Gregory Chow Institute for Studies in Economics, Xiamen University, Xiamen, China; 3 School of Statistics and Mathematics, Central University of Finance and Economics, Beijing, China; 4 National Demonstration Center for Experimental Psychology Education, Faculty of Psychology, Beijing Normal University, Beijing, China; 5 Mental Health Center, Central University of Finance and Economics, Beijing, China; 6 School of Sociology and Psychology, Central University of Finance and Economics, Beijing, China

**Keywords:** childhood maltreatment, latent profile analysis, non-suicidal self-injury, recent stressful life events, self-compassion, self-criticism

## Abstract

**Background:**

Non-suicidal self-injury (NSSI) has increasingly become a common health concern among young adults. To effectively address and prevent NSSI, it is crucial to understand the impact of both early childhood maltreatment and recent stress, which are well-established risk factors. Previous studies have primarily focused on these factors individually or overall, while neglecting the potential heterogeneity of childhood maltreatment and recent stress when these factors co-occur. Therefore, this study aims to investigate the potential latent types of childhood maltreatment and recent stress and their associations with NSSI among young adults.

**Methods:**

A sample of 5,224 young adults were recruited via cluster sampling. Participants completed a battery of self-report questionnaires assessing childhood maltreatment, recent life stress, and NSSI behaviors in a cross-sectional design. Latent profile analysis was conducted to characterize distinct subgroups of childhood maltreatment and recent stress exposure. Subsequently, multiple logistic regression and mediation analyses were utilized to evaluate the differential associations between identified classes and NSSI and to assess indirect associations involving inadequate-self, hated-self, and self-compassion.

**Results:**

Participants’ childhood maltreatment and recent stress were classified into four latent profiles: the “childhood maltreatment predominant group” (*n* = 116), the “stress predominant group” (*n* = 777), the “medium childhood maltreatment and stress group” (*n* = 1,611), and the “low risk group” (*n* = 2,720). Compared to the low risk reference group, the remaining three subgroups showed significantly higher odds of NSSI, with the childhood maltreatment predominant group demonstrating the strongest association (odds ratio = 6.253). Furthermore, self-criticism and self-compassion showed distinct indirect associations between latent profile membership and NSSI.

**Conclusions:**

Young adults demonstrated significant heterogeneity in the joint patterns of childhood maltreatment and recent stress, with certain subgroups being associated with a substantially higher probability of NSSI. Early identification of these at-risk profiles may help mental health professionals and educators to implement tailored assessment and prevention efforts addressing self-related processes, including self-criticism and self-compassion, thereby informing the prevention of NSSI.

## 1 Introduction

Non-suicidal self-injury (NSSI) refers to the deliberate destruction of body tissue without suicidal intent and is recognized as a significant risk factor of subsequent suicidal thoughts and behaviors. Individuals with a history of NSSI are at substantially greater risk for later suicidal ideation and suicide attempts (1). NSSI demonstrates a distinct developmental trajectory, typically emerging during early adolescence and remaining highly prevalent throughout emerging adulthood. A meta-analysis reported prevalence rates of 17.2% among adolescents and 13.4% among university-aged young adults (2), indicating that self-harm and related suicidal behaviors among young adults remain critical public health concerns. Integrated models of NSSI (3) and the Benefits and Barriers Model of NSSI (4) propose that NSSI arises from the interplay of distal stressors (e.g., childhood maltreatment) and proximal stressors (e.g., recent stress). Considering these stressors simultaneously may provide a more comprehensive understanding of how adverse experiences across different developmental periods are associated with NSSI. However, existing NSSI research has rarely examined the joint patterns of childhood maltreatment and recent stress within individuals, relying predominantly on variable-centered approaches. Consequently, examining heterogeneous patterns of childhood maltreatment and recent stress and their associations with NSSI among young adults requires a person-centered perspective.

Self-related negative cognitions, particularly self-criticism and self-punishment, have been identified as important factors contributing to NSSI (5). In contrast, the Benefits and Barriers Model of NSSI suggests that positive self-perceptions and self-compassion may function as important protective factors in associated with lower NSSI risk (4). Consistent with these theoretical perspectives, previous variable-centered research has identified self-criticism as a potential explanatory process between childhood maltreatment and NSSI (6) and reported an indirect association between recent stress and suicidal risk through self-compassion (7). However, whether these processes are involved in the associations between heterogeneous profiles of childhood maltreatment and recent stress and NSSI remains unclear. Accordingly, this study investigates the indirect associations between these profiles and NSSI through self-criticism and self-compassion among Chinese young adults from a person-centered perspective.

### 1.1 Childhood maltreatment, recent stress, and NSSI

Childhood maltreatment refers to abuse and neglect occurring during childhood and is typically operationalized into five core subtypes: sexual abuse, physical abuse, emotional abuse, physical neglect, and emotional neglect. As a pervasive adverse childhood experience (ACE), childhood maltreatment is highly prevalent worldwide, with approximately one-fourth of the adult population has experienced one or more types of childhood maltreatment during childhood (8). Within the NSSI literature, childhood maltreatment is recognized as a well-established distal risk factor. Extant research demonstrates that both global maltreatment scores and specific subtypes—namely emotional, physical, and sexual abuse, alongside physical neglect—significantly predict heightened involvement in NSSI, though empirical evidence for emotional neglect remains less robust (6). Furthermore, cumulative adversity may compound this vulnerability: an investigation into 13 distinct ACEs revealed that high levels of cumulative adversity were robustly associated with NSSI (Odds Ratio [OR] = 5.53), with a larger magnitude of association than that observed for depressive mood (OR = 2.80) and anxiety (OR = 2.05) (9).

In contrast to these distal vulnerabilities, recent stressful life events function as proximal stressors contributing to the occurrence of NSSI. Literature indicates that individuals at risk for NSSI may exhibit intrinsic risk factors, such as heightened stress susceptibility, as well as interpersonal vulnerabilities, including difficulties in social problem-solving and communication (10). When these individuals encounter acute, recent stressors that exceed their adaptive coping capacities, difficulties in emotion regulation may intensify. Consequently, NSSI may function as a maladaptive coping strategy associated with attempts to regulate intense emotional distress, punish the self, or manage interpersonal difficulties.

Although childhood maltreatment and recent stress constitute pivotal risk factors for NSSI and frequently co-occur across the developmental periods, empirical investigations integrating both forms of stress within a single analytical framework remain limited. The cumulative risk hypothesis suggests that psychological risk increases as adverse experiences accumulate, whereas the stress sensitization hypothesis proposes that childhood maltreatment may heighten susceptibility to subsequent stressors (11). Nevertheless, extant NSSI research has predominantly relied on variable-centered approaches, which have yielded inconsistent findings regarding the joint associations of childhood maltreatment and recent stress with negative mental health outcomes. For instance, some studies suggest that recent stressors may strengthen the association between childhood maltreatment and suicidal ideation (12), others indicate that moderate-to-high levels of recent stress may weaken the association between childhood adversity and NSSI (13). These inconsistencies highlight the limitations of variable-centered methods and underscore the need for alternative approaches to capture heterogeneous patterns of childhood maltreatment and recent stress exposure.

Previous research has utilized latent class analysis (LCA) to identify distinct typologies of childhood maltreatment, typically revealing two-to-four-class solutions (14). However, investigations focused exclusively on early adversity cannot capture how childhood maltreatment co-occurs with recent general life stress within individuals. Although a limited line of inquiry has extended LCA to include both childhood abuse and later interpersonal victimization demonstrating associations with anxiety, depression, and PTSD (15, 16). However, these studies examined later interpersonal victimization rather than general recent stress and predominantly involved adult samples, leaving their relevance to NSSI among younger populations unclear. To bridge this empirical gap, the present study employs latent profile analysis (LPA) by incorporating both distal childhood maltreatment and proximal recent stress, thereby elucidating how heterogeneous configurations of these two stress domains associate with NSSI among young adults. We expected profiles characterized by higher levels of both childhood maltreatment and recent stress to show the highest likelihood of NSSI.

### 1.2 The roles of self-criticism and self-compassion

Extant literature indicates that self-criticism mediates the association between childhood emotional abuse and NSSI (6). According to the self-punishment hypothesis (3), individuals who exhibit high levels of self-criticism are more likely to engage in NSSI as a form of self-punishment. Gilbert and colleagues conceptualize self-criticism as a response to frustration or disappointment, delineating two forms: inadequate-self, characterized by feelings of personal inadequacy and a tendency to dwell on mistakes, and the more severe hated-self, characterized by intense self-dislike and self-directed hostility (17). Although both forms of self-criticism may increase vulnerability to NSSI, hated-self may be particularly harmful because it involves more intense self-directed hostility and has shown stronger associations with psychopathological symptoms (17). Relatedly, self-punishment cognitions have been found to distinguish individuals with and without a history of NSSI (18).

In contrast to self-criticism, self-compassion refers to kind, caring, and supportive inner responses toward oneself when facing setbacks (19) and closely parallels self-reassurance in Gilbert et al.’s (17). Self-compassion has been identified as a promising protective factor against NSSI and is believed to develop during early childhood (20). In related research, self-compassion has also been found to mediate the relationship between negative life events and suicidal risk, providing further support for its potential protective role in stress-related pathways (7). Although related, self-criticism and self-compassion are not simply opposite ends of a single continuum. Psychometric research distinguishes self-compassionate responding from self-uncompassionate responding, while neuroimaging evidence differentiates self-criticism from self-reassurance (21, 22).

The Benefits and Barriers Model of NSSI provides further theoretical justification for considering both processes. Self-criticism, characterized by negative self-evaluations, may erode the positive self-view barrier to NSSI (23). Extending this framework, self-compassion may represent a complementary protective process by fostering positive and supportive self-relating (24). Accordingly, some scholars have argued that self-criticism and self-compassion should be examined simultaneously, as they represent transdiagnostic vulnerability and protective factors, respectively (25). Despite increasing recognition of their importance, little is known about how self-criticism and self-compassion operate under the combined influence of distal stressors (e.g., childhood maltreatment) and proximal stressors (e.g., recent stress). Moreover, whether these self-related processes explain the association between heterogeneous profiles of childhood maltreatment and recent stress and NSSI remains unclear. Therefore, the present study examines the indirect associations between profiles of childhood maltreatment and recent stress and NSSI through self-criticism and self-compassion.

### 1.3 Aim of this study

Although previous studies have examined the associations of childhood maltreatment and recent stress with NSSI, these adverse experiences have largely been investigated separately or from an additive perspective. Relatively little is known about how childhood maltreatment and recent stress co-occur within individuals and whether distinct patterns of exposure across these two developmental periods are differentially associated with NSSI. By integrating the perspectives of distal childhood adversity and proximal recent stress within a person-centered framework, the present study sought to provide a more nuanced understanding of heterogeneous patterns of exposure and their associations with NSSI. The present study had three primary objectives. First, it sought to identify distinct latent profiles based on experiences of childhood maltreatment and recent stress to characterize the joint exposure pattern represented by each profile. Second, it examined the associations between profile membership and NSSI. Third, it investigated the indirect associations between profile membership and NSSI through inadequate-self, hated-self, and self-compassion (operationalized as reassured-self). Accordingly, we hypothesized that: (H1) distinct profiles of childhood maltreatment and recent stress would emerge; (H2) profiles characterized by higher levels of childhood maltreatment and/or recent stress would be associated with a higher probability of NSSI; and (H3) these profiles would show significant indirect associations with NSSI through higher inadequate-self and hated-self and lower self-compassion.

## 2 Methods

### 2.1 Participants and procedure

This cross-sectional study recruited young adults from a comprehensive university in Beijing, China, during October and November, 2021. Participants were recruited using a cluster sampling approach. The online questionnaire link was distributed to students through their student counselors, who assisted in disseminating the survey within their respective classes. Before beginning the survey, participants provided electronic informed consent, and were informed that participation was voluntary, anonymous, and that they could withdraw at any time without penalty. Completion of the questionnaire had no impact on students’ academic standing or access to university services. Participants who completed the survey received a small gift as a token of appreciation for their participation. Ethical approval for the study was obtained from the Ethics Committee of [MASKED FOR REVIEW].

The inclusion criterion was age between 18 and 50 years, consistent with the definition of young adults (26). Exclusion criteria included: (1) an average response time of less than 1 second per item and (2) failure to pass the attention-check question. A total of 6,319 questionnaires were distributed, and 5,224 valid responses were retained for analysis, yielding a valid response rate of 82.67%.

### 2.2 Measurements

#### 2.2.1 Sociodemographic variables

The questionnaire included six questions on sociodemographic information, covering age, sex (biological sex assigned at birth), type of residence (city, town or country), whether the respondent is an only child, parents’ marital status, and financial status (whether confronting financial difficulty or not). All sociodemographic variables were coded as categorical variables (binary or multicategory) except for age.

#### 2.2.2 Childhood maltreatment

The Childhood Trauma Questionnaire–28 item Short Form (CTQ-SF; (27)) was used to measure childhood trauma experienced before the age of 16. Zhao and colleagues adapted and revised the Chinese version of the questionnaire (28). The CTQ-SF includes five subscales—emotional abuse, physical abuse, emotional neglect, physical neglect, and sexual abuse—as well as three validity items, totaling 28 items. Each item is rated on a 5-point scale, ranging from 0 (“*Never*”) to 4 (“*Always*”), with higher scores indicating more severe childhood maltreatment experiences. The Chinese revised version of the CTQ-SF had demonstrated good reliability and validity (29, 30). In the present study, the Cronbach’s *α* coefficients for the subscales ranged from 0.519 to 0.764 while the Cronbach’s *α* for the overall CTQ-SF was 0.820.

#### 2.2.3 Recent life stress

The revised version of the Adolescent Self-Rating Life Events Check (ASLEC) originally developed by (31), as modified by (32) was used to assess recent life stress. The ASLEC consists of 25 items encompassing five dimensions: punishment stress, loss stress, interpersonal stress, academic stress, and adaptation stress. Participants were asked to indicate whether each event had occurred within the past 12 months to themselves or their families. If an event occurred, participants then rated its impact on a six-point scale, ranging from 0 (“*not occurred*”) to 5 (“*extremely severe impact*”). Higher scores reflect a greater negative impact of these events on the individual. In the present study, the overall Cronbach’s *α* for the ASLEC was 0.952. The Cronbach’s *α* coefficients for the five subscales ranged from 0.706 to 0.928.

#### 2.2.4 Non-suicidal self-injury

The Adolescent Non-Suicidal Self-Injury Assessment Questionnaire (ANSAQ), developed by Wan Yuhui et al., was used to assess the occurrence of NSSI among university students within the past year (33). The ANSAQ comprises two parts: a behavior questionnaire and a function questionnaire. The behavior questionnaire consists of 12 items representing various NSSI behaviors (e.g., pinching, scratching, pulling one’s hair, cutting, burning, carving words or symbols onto the skin). The function questionnaire contains 19 items divided into three dimensions: (1) emotional expression (4 items), measuring the expression of emotional feelings; (2) self-negative reinforcement (5 items), assessing behaviors aimed at relieving or escaping undesirable states; and (3) Prosocial (10 items), reflecting NSSI behaviors conducted to create a positive state or fulfill social needs. The behavioral items included in this instrument closely mirror those used in the Functional Assessment of Self-Mutilation (FASM) and Inventory of Statements About Self-Injury (ISAS), ensuring comparability with widely used international assessments of NSSI. This consistency makes it a suitable and reliable measure for evaluating NSSI behaviors in Chinese populations. Because this study focuses on the impact of childhood maltreatment and recent stress on NSSI, only the first 12 items of the behavior questionnaire were considered to calculate the frequency of NSSI behaviors, while the functional aspects of NSSI were not included in the analysis. In the present study, using these 12 items yielded an overall Cronbach’s *α* of 0.845.

Furthermore, NSSI occurrence was used as the primary outcome variable in the present study. NSSI occurrence was defined as the presence of any self-injurious behavior among the 12 NSSI behaviors assessed by the questionnaire. Specifically, participants who reported engaging in at least one type of NSSI behavior were classified as having NSSI occurrence, whereas those who reported no NSSI behaviors were classified as having no NSSI occurrence. This operationalization has been commonly adopted in previous studies using similar questionnaires to assess NSSI behaviors (34, 35).

#### 2.2.5 Self-criticism and self-compassion

This study adopted the Forms of Self-Criticizing/Attacking and Self-Reassuring Scale (FSCRS) (17), a 22-item self-rating instrument designed to assess self-criticism and the capacity for self-reassurance in response to setbacks and failures. The scale comprises three subscales: inadequate-self (9 items), which assesses feelings of personal inadequacy and a tendency to dwell on mistakes; hated-self (5 items), which assesses a more severe form of self-criticism characterized by intense self-dislike and self-directed hostility; and reassured-self (8 items), which assesses the ability to reassure, encourage, and support oneself when facing setbacks. In the present study, reassured-self was used as an operational indicator of self-compassion because it captures warm and supportive responses toward the self. Items are rated on a 5-point Likert scale ranging from 0 (not like me at all) to 4 (extremely like me), with higher scores indicating a stronger sense of inadequacy, self-hate, or self-compassion, respectively. The FSCRS has been validated in 13 countries and regions, demonstrating excellent reliability and validity (36). In the present study, the overall Cronbach’s *α* coefficients for the FSCRS and its subscales ranged from 0.892 to 0.917.

### 2.3 Analytic approach

Data analyses were all conducted using SPSS 25.0, R 4.3.1 and Mplus 8.3. Specifically, SPSS was used for descriptive and inferential statistical analyses, R was used for data cleaning and preprocessing, and Mplus was used for latent profile analysis (LPA) and mediation analyses. Firstly, the characteristics of the main variable concerned in the study were explored. Descriptive statistics of sociodemographic variables and NSSI behavior were analyzed. Notably, we observed that some main variables exhibited a certain degree of skewness and kurtosis, which led us to adopt some corrective methods in the subsequent analysis. For instance, Spearman’s rank correlation analyzes were employed to examine the relationships among the variables. A series of supplementary analyses suggested that common method bias was unlikely to substantially influence the statistical analyses reported in the present study (*see* **Supplementary Materials**).

Secondly, LPA was conducted to identify the latent structures of CM. The following indicators were used as references for profile selection: information criteria, likelihood ratio test, and entropy. Information criteria include Akaike’s information criterion (AIC), Bayesian information criterion (BIC), and sample size-adjusted BIC (aBIC). Smaller values of these indices indicate better model fit (37). The Vuong-Lo-Mendell-Rubin likelihood ratio test (LMR) and the parametric bootstrapped likelihood ratio test (BLRT) were also considered. Significant p-values on these tests indicate that a model with k profiles fits significantly better than a model with k-1 profiles (38). The entropy index ranges from 0 to 1, with values closer to 1 indicating more accurate classification. An entropy of approximately 0.8 suggests a classification accuracy exceeding 90% (39). In addition to these statistical criteria, theoretical rationality and practical interpretability were considered when deciding on the final number of profiles. Based on theoretical and practical considerations, names were assigned to each profile. To reduce multicollinearity and facilitate interpretation, all continuous variables were standardized prior to conducting the LPA.

Thirdly, after determining the optimal LPA model, One-way ANOVA was used to examine whether demographic variables differed significantly among different profiles. Concerning the skewness of childhood maltreatment and recent stress dimensions, the Kruskal-Wallis H test was used to compare differences among latent profiles. Pairwise comparisons between profiles were adjusted for significance using the Bonferroni correction.

Fourthly, for the purpose of examining the influence of different latent profiles on NSSI, a binary logistic regression model was constructed using NSSI occurrence (0 = “*no*”, 1 = “*yes*”) as the dependent variable, and latent profile dummy variables as the independent variables. For example, if the low risk (LR) group served as the reference category, then the three dummy variables represented the other three profiles: childhood maltreatment predominant (CMP) group, stress predominant (SP) group, medium childhood maltreatment & stress (MCMS) group).

Finally, a series of mediation analyses were built to investigate the mediation of self-criticism (inadequate-self and hated-self) and self-compassion between the relationship of different profiles and NSSI. The mediating effects were examined using parallel mediation models with a multi-categorical independent variable. In each mediation model, inadequate-self and hated-self and self-compassion served as three parallel mediating variables, and NSSI occurrence as the dependent variable. Similar to the logistic regression, one profile served as a reference, and three dummy variables served as independent variables, which were created by using indicator coding to represent the compared profiles (40). To examine differences between any two of the four profiles, different profiles were specified as the reference category in separate mediation analyses. Specifically, the results were presented for three profile comparisons using the LR group as the reference category, two using the MCMS group as the reference category, and one using the SP group as the reference category, thereby enabling pairwise comparisons among all latent profiles.

Since all models were saturated models, model fit indices were not applicable in the present study. Because NSSI occurrence was a binary outcome, the mediation analysis was not conducted using ordinary linear path analysis. Instead, a categorical structural equation modeling approach was implemented, with NSSI occurrence specified as a categorical dependent variable and estimated using a probit link function with the weighted least squares mean and variance adjusted (WLSMV) estimator. Indirect effects were estimated using the product-of-coefficients approach, in which the indirect effect was calculated as the product of the path coefficient from latent profile membership to the mediator and the path coefficient from the mediator to NSSI occurrence. Bootstrap confidence intervals were estimated based on 5,000 bootstrap replications. An indirect effect was considered statistically significant if the 95% confidence interval did not include zero.

In both the logistic regression and mediation analyses, sex, age, only-child status, and financial status were included as covariates, as these demographic variables have been widely controlled for in previous studies examining self-harm and related psychological outcomes (41, 42). In the mediation models, these covariates controlled both the three mediators and the NSSI outcome.

## 3 Results

### 3.1 Demographic characteristics and correlations between main variables

**Figure 1:**
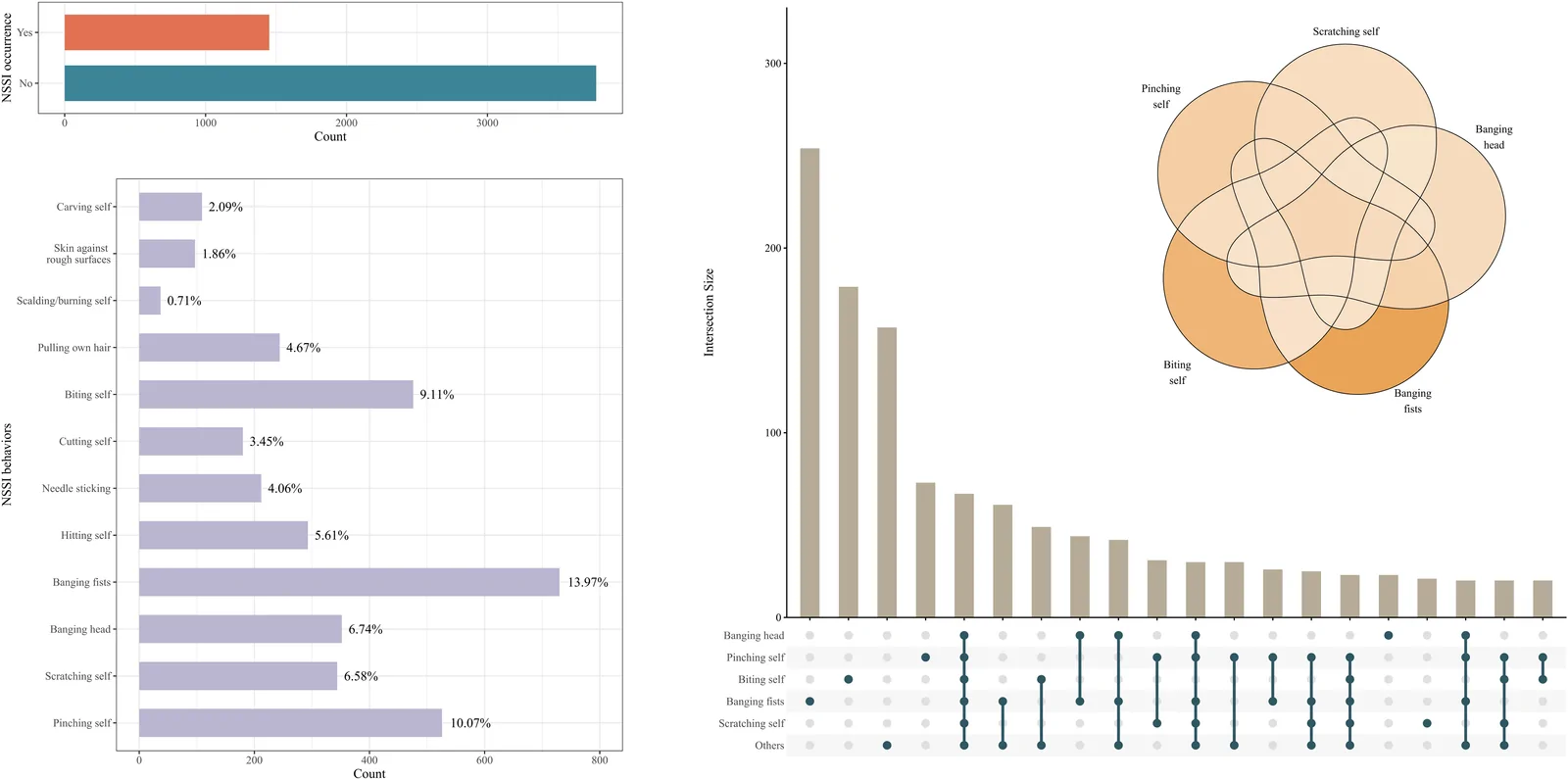
Characteristics of NSSI among participants (*N* = 5,224). The top-left panel shows the overall prevalence of NSSI in the full sample; the bottom-left panel presents the number of occurrences for each specific NSSI behavior; the right panel illustrates the five most frequently occurring NSSI behaviors and the overlaps among behavior subsets. In the right panel, “Banging head” and “Banging fists” are abbreviated terms referring to the following two NSSI behaviors, respectively: “Banging the head against hard objects” and “Banging the fists against hard objects”.

Among all participants (*N* = 5,224), 3,617 (69.24%) were female, with an average age of 20.96 ± 3.44 years (range = 18–44 years). The majority of the participants were from urban areas (50.45%), had siblings (48.24%), were from intact (first-marriage) families (80.78%), and reported no financial difficulties (67.44%). Regarding the behavior of NSSI, 1,452 individuals (27.79%) reported having engaged in NSSI within the past year. Among the individuals who had engaged in NSSI, the most common form of NSSI was intentionally striking hard surfaces (such as walls, desks, windows, or floors) with one’s fist (50.28%), followed by intentionally pinching oneself (36.23%) (*see* **Figure 1**).

An independent-samples t-test was conducted to examine whether age differed between participants with and without NSSI occurrence. The results showed that the age of participants with NSSI occurrence (20.02 ± 2.91) was significantly different from those without NSSI occurrence (21.31 ± 3.55) (*t* = 12.44, *p* = .000).

Chi-square tests were conducted to examine differences in NSSI occurrence across various demographic variables. As the proportion of missing values for demographic variables did not exceed 10% and no imputation could be performed, participants with missing values on the corresponding variables were excluded from the respective chi-square analyses. The results indicated that sex and whether the participant was an only child had significant effects on NSSI occurrence. In contrast, variables such as place of residence, family financial status, and parents’ marital status showed no significant differences (*see* **Table 1**).

**Table 1:** NSSI occurrence across various demographic variables (*N* = 5,224).

Variables	*n* (%)	NSSI			
		Occurred *n* (%)	Not occurred *n* (%)	*χ* ^2^	*p*
Sex				41.562***	.000
Male	1607 (30.76%)	543 (33.79%)	1064 (66.21%)		
Female	3617 (69.24%)	909 (25.13%)	2708 (74.87%)		
Type of residence				1.227	.541
City	2372 (50.45%)	670 (28.25%)	1702 (71.75%)		
Town	1252 (26.63%)	328 (26.20%)	924 (73.80%)		
Country	1078 (22.93%)	268 (24.86%)	810 (75.14%)		
Only child				9.721**	.002
Yes	2198 (42.08%)	640 (29.12%)	1558 (70.88%)		
No	2520 (48.24%)	632 (25.08%)	1888 (74.92%)		
Parents’ marital status				1.138	.768
First marriage	4220 (80.78%)	1135 (26.90%)	3085 (73.10%)		
Divorced	307 (5.88%)	79 (25.73%)	228 (74.27%)		
Remarried	90 (1.72%)	28 (31.11%)	62 (68.89%)		
Others	87 (1.67%)	22 (25.29%)	65 (74.71%)		
Financial Status				1.978	.160
Difficulties	1177 (22.53%)	299 (25.40%)	878 (74.60%)		
No difficulties	3523 (67.44%)	969 (27.50%)	2554 (72.50%)		

NSSI refers to non-suicidal self-injury. The sum of the categories for some demographic variables in the second column does not equal the total sample size (*N* = 5,224) due to missing data in these variables. Participants with missing values on the corresponding variables were excluded from the respective chi-square analyses. **p < 0.01, ***p < 0.001.

The results of the correlation analyses indicated that all dimensions of childhood maltreatment and recent stress were significantly positively associated with NSSI occurrence. Furthermore, both inadequate-self and hated-self exhibited significant positive correlations with childhood maltreatment, the various dimensions of recent stress, and NSSI occurrence. In contrast, self-compassion showed significant negative correlations with all of the aforementioned variables (*see* **Supplementary Figure 1**).

### 3.2 Latent profiles and profile characteristics of childhood maltreatment and recent stress

To explore latent profile types of childhood maltreatment and recent stress among young adults, each dimension’s score was first converted to a standardized score (For descriptive statistics of each dimension before standardizing, *see* **Supplementary Table 1**). Using the scores of ten dimensions, models with one to five latent profiles were estimated in sequence.

The results showed that the AIC and BIC values decreased as the number of profiles increased, and the entropy values were all above.90, indicating effective classification (*see* **Table 2**). Model comparisons revealed that the five-class solution had a non-significant LMR test (*p* = 0.332), suggesting no significant improvement on model fit from the four-class solution to the five-class solution.

**Table 2:** Model fit information for latent profile analysis.

Classes	1	2	3	4	5
AIC	148280.697	133994.781	128709.127	124063.242	121729.776
BIC	148411.917	134198.172	128984.689	124410.976	122149.682
aBIC	148348.364	134099.664	128851.227	124242.560	121946.311
Entropy		0.959	0.974	0.901	0.913
LMR(*p*)		.000	.011	.000	.201
BLRT(*p*)		.000	.000	.000	.000

**Figure 2:**
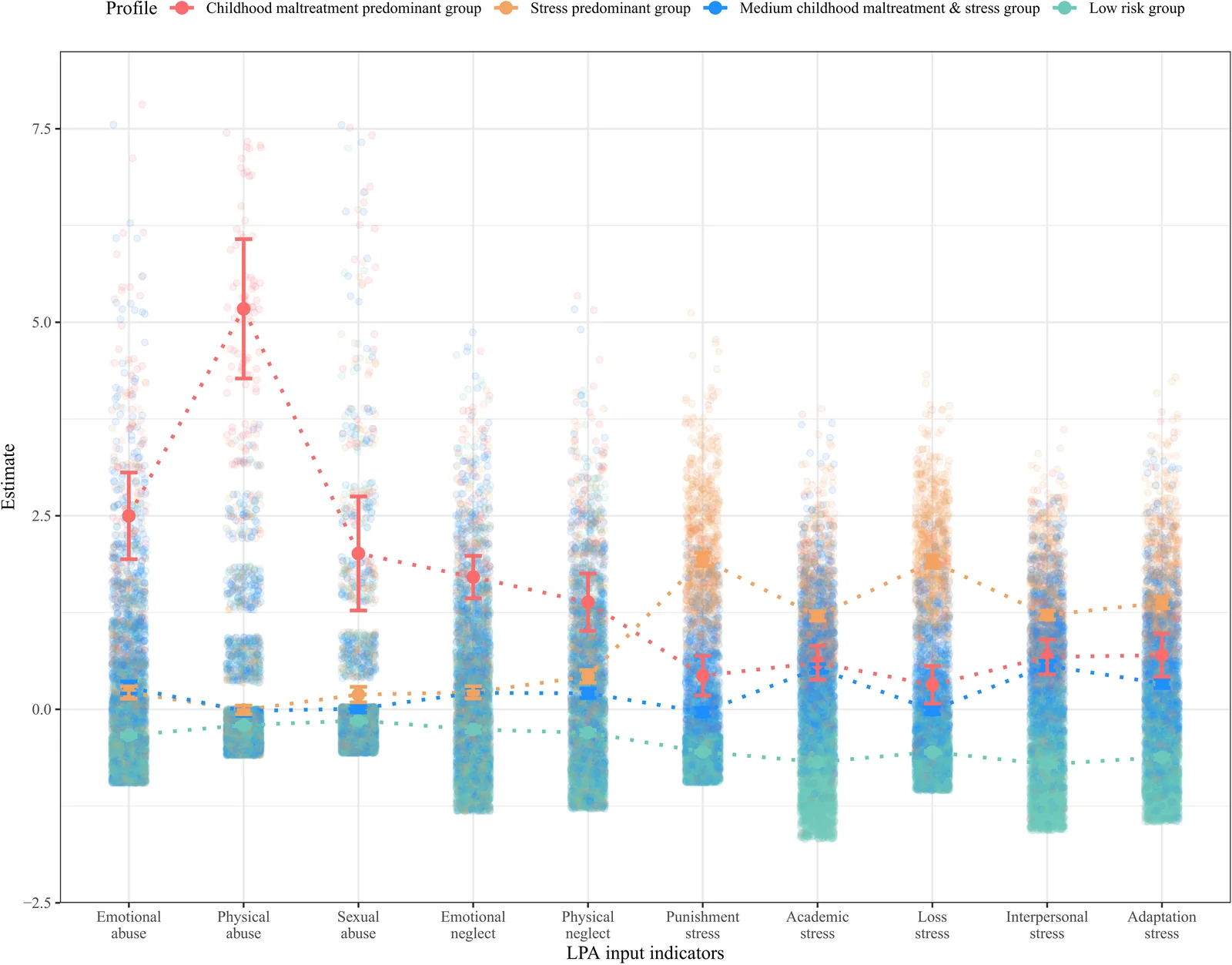
Four latent profiles of childhood maltreatment and recent stress. In latent profile analysis, each dimension’s score was converted to a standardized score.

Based on theoretical considerations, the four-class solution demonstrated four distinct and interpretable subgroups (*see* **Figure 2**). Compared with the three-class solution (*see* **Supplementary Figure 2A**), the four-class solution additionally identified a medium childhood maltreatment and stress group. This subgroup reflects the co-occurrence of moderate childhood maltreatment and recent stress, which could be overlooked when young adults are classified only into the low-risk, childhood maltreatment-predominant, and stress-predominant groups. Its identification is theoretically and clinically meaningful because the accumulation of moderate adversity across both domains may require targeted prevention and intervention. In contrast, the five-class solution mainly further divided the childhood maltreatment-predominant group into profiles with similar patterns but different severity levels, without identifying a substantively distinct configuration (*see* **Supplementary Figure 2B**). Therefore, the four-class solution provided the best balance between conceptual interpretability and parsimony.

Furthermore, the classification quality of the four-class solution was supported by high average posterior probabilities and low classification error rates (*see* **Table 3**). Specifically, all classes showed average posterior probabilities greater than 0.90, indicating excellent classification accuracy. A series of sensitivity analyses further supported the appropriateness and robustness of the four-class solution (*see* **Supplementary Materials**). Taken together, the four-class solution demonstrated clear empirical separation, strong statistical reliability, and meaningful theoretical interpretability. Therefore, the four-class solution was selected as the final model in the present study.

**Table 3:** Profile composition and classification quality of the four-class model.

Group	n	Proportion (%)	Average posterior probability	Classification error rate
CMP	116	2.22%	0.976	0.024
SP	777	14.87%	0.974	0.026
MCMS	1611	30.84%	0.906	0.094
LR	2720	52.07%	0.953	0.047

LR, low-risk group; CMP, childhood maltreatment predominant group; SP, stress predominant group; MCMS, medium childhood maltreatment and stress group.

As shown in the figure (*see* **Figure 2**), the first profile was characterized by high scores on childhood maltreatment measures and was named the childhood maltreatment predominant group (CMP) (2.22%). The second profile exhibited relatively high levels of recent stress and was named the stress predominant group (SP) (14.87%). The third profile showed moderate scores on both childhood maltreatment and recent stress dimensions and was termed the medium childhood maltreatment & stress group (MCMS) (30.84%). The fourth profile demonstrated low scores across both childhood maltreatment and recent stress dimensions and was labeled the low risk group (LR) (52.07%).

Differences in the scores of childhood maltreatment and recent stress dimensions across the latent profiles were analyzed (*see* **Supplementary Table 4**). Since the distributions of childhood maltreatment and recent stress dimensions in this study deviated from normality, the Kruskal-Wallis *H* test was employed for group comparisons among the four latent profiles. Pairwise comparisons were adjusted for significance using the Bonferroni correction. Overall, the results revealed that the mean scores across all ten dimensions differed significantly among the identified profiles (all *p* <.001). Multiple comparison analyses further indicated that, across the five dimensions of childhood maltreatment, the CMP group exhibited higher overall mean levels than the SP group (mean difference = 1.911 ~ 5.543, all *p* <.001). In contrast, the SP group showed significantly higher mean levels of recent stress than the CMP group (mean difference = 2.194 ~ 9.737, all *p* <.01), suggesting that the CMP and SP groups were appropriately differentiated based on their distinct profile characteristics. The MCMS group demonstrated substantially lower levels of childhood maltreatment than the CMP group (mean difference = 2.099 ~ 5.558, all *p* <.001) and lower levels of recent stress than the SP group (mean difference = 2.590 ~ 12.503, all *p* <.001). Furthermore, the LR group showed significantly lower levels across all ten dimensions compared with the MCMS group (mean difference = 0.154 ~ 5.931, all *p* <.001).

### 3.3 The association between CM profiles and NSSI

**Figure 3:**
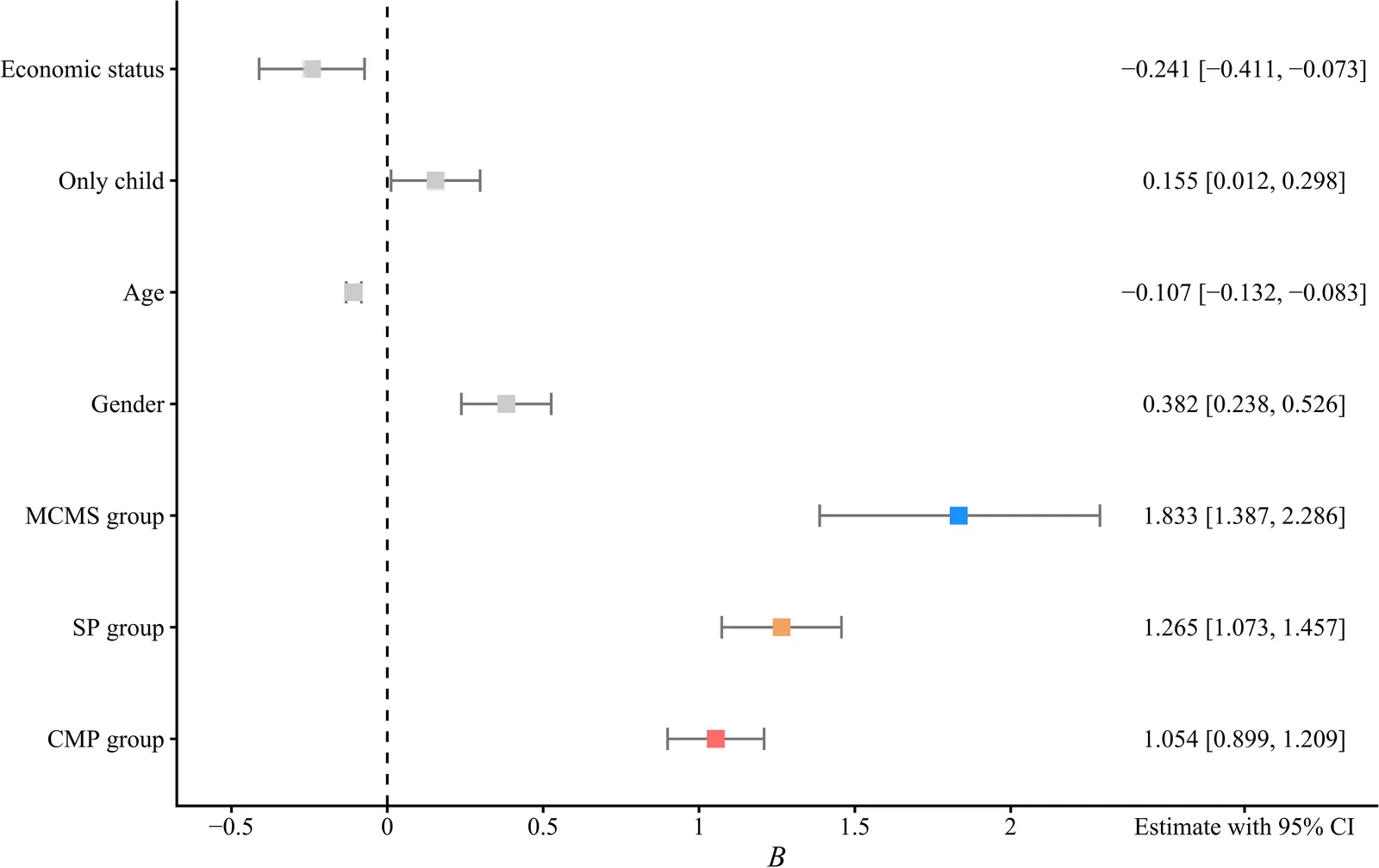
Logistic regression of latent profiles on NSSI. The estimates presented in the figure are log-odds coefficients, which represent the regression coefficients from the logistic regression model. The low-risk group served as the reference category. CM, childhood maltreatment. Sex, age, only-child and financial status were included as covariates, coded as follows: male = 0, female = 1; only child = 1, not only child = 0; financial difficulty = 1, no financial difficulty = 0.

The results of the binary logistic regression indicated that, relative to the LR group, all three other profiles were associated with an increased probability of NSSI occurrence (*see* **Figure 3**). In particular, participants in the CMP group showed significantly higher odds of NSSI occurrence compared with those in the LR group (OR = 6.253, 95% CI = [3.994, 9.790], *p* <.001).

In addition, to further characterize the relative differences in NSSI occurrence among latent profiles, we conducted three additional logistic regression models using each of the other profiles (CMP, SP, and MCMS) as the reference category (*see* **Supplementary Table 5**). These analyses allowed for direct comparisons of NSSI occurrence odds between different profiles. The results demonstrated a clear gradient pattern across profiles, with the CMP group showing the highest odds of NSSI occurrence, followed by the SP group, the MCMS group, and the LR group. Specifically, the CMP group showed significantly higher odds of NSSI occurrence than the SP group (OR = 1.613, 95% CI = [1.115, 2.796], *p* <.001), the SP group showed higher odds than the MCMS group (OR = 1.235, 95% CI = [1.021, 1.494], *p* <.001), and the MCMS group showed higher odds than the LR group (OR = 2.869, 95% CI = [2.457, 3.350], *p* <.001).

### 3.4 Mediating role of self-criticism and self-compassion

**Figure 4:**
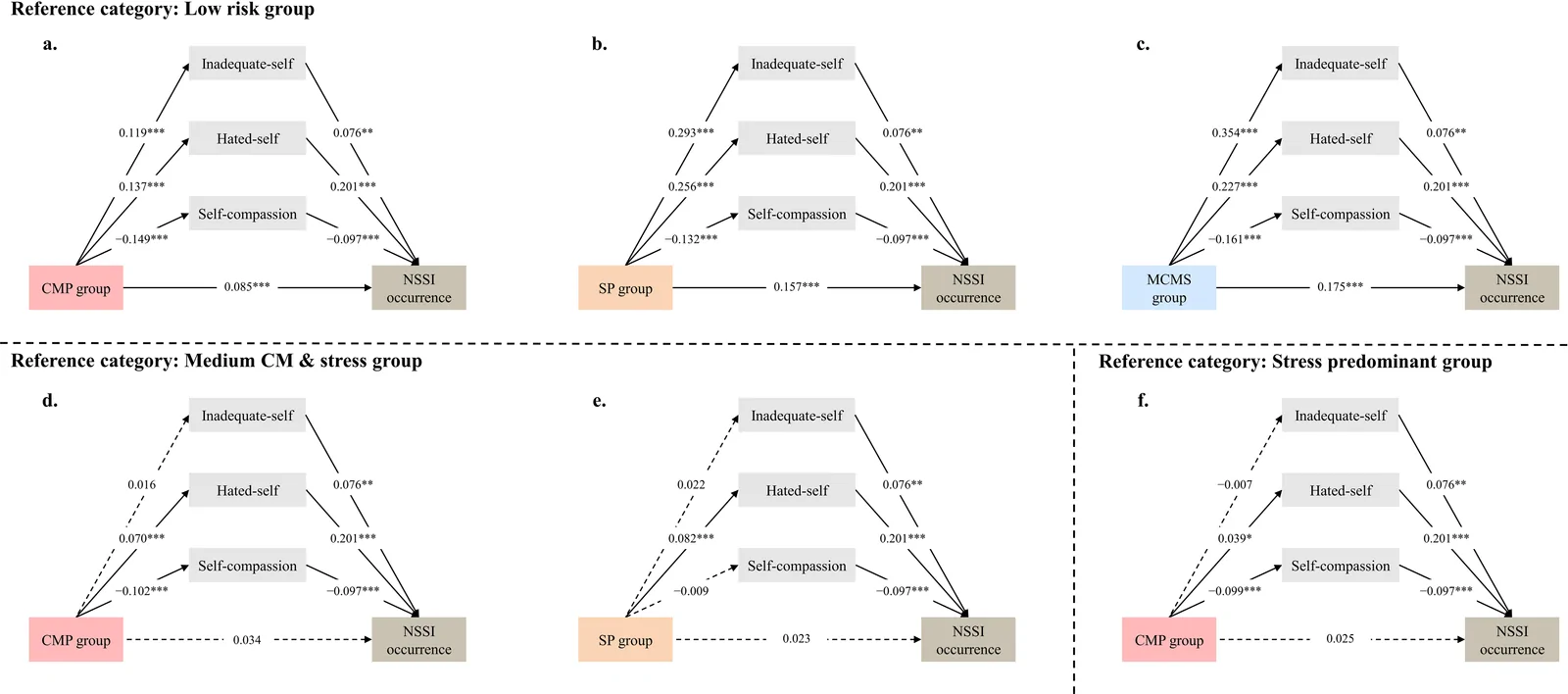
Multicategorical mediation analyses examining pairwise differences among latent profiles. **(A)** LR group vs. CMP group; **(B)** LR group vs. SP group; **(C)** LR group vs. MCMS group; **(D)** MCMS group vs. CMP group; **(E)** MCMS group vs. SP group; **(F)** SP group vs. CMP group. This figure presented the path coefficients estimated from the multicategorical mediation model. In each analysis, one latent profile served as the reference group, and the other three profiles were represented by dummy-coded variables as independent variables to estimate their associations with the mediators and NSSI occurrence. Each panel displayed the path coefficients corresponding to a specific pairwise comparison between latent profiles, including the associations of profile membership with the mediators and NSSI occurrence, as well as the associations between the mediators and NSSI occurrence. LR, low-risk group; CMP, childhood maltreatment predominant group; SP, stress predominant group; MCMS, medium childhood maltreatment & stress group. Sex, age, only-child and financial status were included as covariates, coded as follows: male = 0, female = 1; only child = 1, not only child = 0; financial difficulty = 1, no financial difficulty = 0. In the final model, correlations were specified among self-criticism, hated-self, and self-compassion. *p < 0.05, **p < 0.01, ***p < 0.001.

Six mediation models were conducted to examine whether the differences among latent profiles could be explained by the mediating effects of self-criticism and self-compassion (*see* **Figure 4**). The specific path coefficients and effect sizes of the mediation models are presented in the **Supplementary Materials** (*see* **Supplementary Table 6**).

The results showed that with the LR group set as the reference group (*see* **Figures 4A–C**), the results indicated that MCMS, SP and CMP group were all positively associated with inadequate-self and hated-self (*β* = 0.119 ~ 0.354, all *p* <.001), both of which were associated with a higher probability of NSSI occurrence (*β* = 0.076 ~ 0.201, all *p* <.01). Correspondingly, all three groups were negatively associated with self-compassion (*β* = −0.161 ~ −0.132, all *p* <.001), which projected lower probability of NSSI occurrence (*β* = −0.097, *p* <.001).

While MCMS group served as reference (*see* **Figures 4D, E**), the results indicated the relative indirect effects of both SP and CMP group via inadequate-self on NSSI occurrence were nonsignificant (indirect effect = 0.001 ~ 0.002, all *p* > 0.05), whereas the relative indirect effects via hated-self were significant (indirect effect = 0.014 ~ 0.017, all *p* < 0.001), and both groups were positively associated with hated-self (*β* = 0.070 ~ 0.082, all *p* <.001). The mediating effects via self-compassion differed between SP and CMP group. Relative to MCMS group, CMP group was negatively associated with self-compassion (*β* = −0.102, *p* <.001), whereas the SP group was not significant on this path (*β* = −0.009, *p* >.05).

The discrepancy of mediating effects across SP and CMP group was contrasted by setting SP group as the reference group (*see* **Figure 4F**). The results revealed that CMP group was positively associated with hated-self (*β* = 0.039, *p* <.05), and negatively associated with self-compassion (*β* = −0.099, *p* <.001). Meanwhile, the relative indirect effects of inadequate-self were non-significant (*β* = −0.007, *p* >.05).

## 4 Discussion

Using LPA in a large sample, the present study identified distinct patterns of childhood maltreatment and recent stress exposure among Chinese young adults. Furthermore, it examined the associations between different profiles of childhood maltreatment combined with recent stress and NSSI, as well as the potential psychological mechanisms underlying these associations.

The analysis identified four distinct latent profiles, demonstrating marked heterogeneity in the latent profiles of childhood maltreatment and recent stress among Chinese young adults. This finding corroborated our first hypothesis, indicating that stress exposure manifests marked temporal variation across childhood and young adult developmental epochs. Beyond the LR group—which exhibited consistently low stress exposure across both stages and comprised 52% of the sample—three other distinct stress patterns emerged: CMP, SP, and MCMS. Notably, the SP and MCMS profiles exemplify patterns of childhood maltreatment and recent stress. Whereas previous studies have primarily examined the co-occurrence of childhood and later victimization (15, 16), the present findings extend this literature to broader recent stress exposure. Specifically, the SP profile indicated that recent stress may occur without elevated childhood maltreatment, whereas the MCMS profile reflected the co-occurrence of relatively moderate levels of childhood maltreatment and recent stress. These results are relevant to the stress susceptibility hypothesis (11, 43).

Contrary to initial expectations, no latent profile simultaneously characterized by high childhood maltreatment and high recent stress was identified. Instead, the CMP group exhibited only moderate levels of recent stress, while the SP group showed high recent stress but only moderate childhood maltreatment. Both profiles showed the strongest associations with NSSI. This pattern differs from the “high + high” group identified in previous research (16), possibly because that research focused primarily on victimization across developmental periods, whereas the present study included broader forms of recent stress. Consistent with prior variable-centered research showing that moderate-to-high recent stress attenuates the association between childhood maltreatment and NSSI (13), the present pattern may indicate a potential threshold or saturation effect, beyond which childhood maltreatment and recent stress no longer amplify each other’s effects. Moreover, the absence of a “high + high” profile illustrates the value of person-centered approaches in complementing variable-centered approach by identifying naturally occurring configurations of adversity across developmental periods (44). These findings underscore the importance of jointly assessing childhood maltreatment and recent stress in clinical assessment.

After identifying four distinct latent profiles, this study evaluated their respective concurrent associations with NSSI. With the LR group designated as the reference category, all other profiles were associated with a higher probability of NSSI. The CMP group, in particular, demonstrated a notably higher risk of NSSI compared to the remaining groups, a finding congruent with previous observations (16). Moreover, the CMP group exhibited elevated levels of multiple forms of childhood maltreatment. This finding aligns with extensive literature indicating that various types of early adversity frequently co-occur and are collectively associated with cumulative adverse psychological outcomes, including depression, post-traumatic stress symptoms, anxiety, and suicidal behaviors (9, 15, 45, 46). Given that the CMP profile showed the strongest concurrent association with NSSI, prioritized institutional attention and targeted allocation of clinical resources to this specific subpopulation may be warranted.

Furthermore, by simultaneously examining the indirect associations through self-criticism (i.e., inadequate-self and hated-self) and self-compassion (operationalized as reassured-self), this study compared their relative contributions to the association between childhood maltreatment and recent stress profiles and NSSI. Using the LR group as the reference, all three higher-risk groups (CMP, SP, and MCMS) showed indirect associations with a higher probability of NSSI through higher levels of inadequate-self and hated-self, as well as lower levels of self-compassion. These findings are consistent with Nock’s self-punishment hypothesis from a person-centered perspective and extend previous variable-centered evidence regarding the indirect associations of self-criticism and self-compassion with adversity and NSSI (3, 6). Notably, self-compassion consistently showed a protective indirect association across profiles, highlighting its potential protective role in relation to NSSI (47). Taken together, these findings are consistent with Gilbert’s theoretical framework, in which self-criticism represents a threat-related way of relating to oneself, whereas self-reassurance and compassion involve more supportive self-relating associated with the Soothing-Safeness system (48, 49).

Further analyses revealed that hated-self was the most consistent variable accounting for the relative indirect associations across profile comparisons. Relative to the MCMS group, significant indirect associations between the CMP group and NSSI were observed through hated-self and self-compassion, whereas the association between the SP group and NSSI occurred indirectly only through hated-self. A similar pattern emerged when the SP group served as the reference category, with hated-self and self-compassion again accounting for the indirect association between the CMP group and NSSI. These findings suggest that hated-self may represent the most robust self-related correlate of the indirect associations between profiles of childhood maltreatment and recent stress and NSSI. One possible theoretical interpretation is that hated-self represents a more severe threat-related form of self-criticism. Repeated exposure to adversity may strengthen threat-related cognitive and emotional responses, resulting in sensitized pathways that become increasingly accessible over time and heighten vulnerability to NSSI (50). Consistent with this interpretation, hated-self is strongly associated with self-directed disgust, self-rejection, and destructive impulses, making it particularly pathogenic in the context of self-injurious behavior (17). Ecological momentary assessment studies further indicate that trait-level self-punishment, rather than momentary self-criticism, predicts NSSI urges (18). In contrast, inadequate-self did not show a consistent indirect association when comparing different profiles of childhood maltreatment and recent stress with NSSI. One possible explanation is that inadequate-self is comparatively less pathogenic than hated-self (17) and may coexist with self-corrective functions that can be adaptive in some contexts (51).

When self-compassion was examined, the indirect association between the SP group and NSSI through self-compassion was not significant when the MCMS group served as the reference category. This finding suggests that elevated recent stress in the absence of high childhood maltreatment is less strongly associated with self-compassion than differences between profiles involving childhood maltreatment. In contrast, self-compassion showed consistent protective indirect associations in comparisons involving differences in childhood maltreatment exposure. This pattern may reflect the relatively stable and enduring nature of self-compassion as a self-regulatory resource, which may be more strongly shaped by distal developmental experiences such as childhood maltreatment (52).

The present findings have important theoretical and practical implications. From a theoretical perspective, the identification of four distinct childhood maltreatment and recent stress profiles is relevant to the cumulative risk hypothesis and the stress susceptibility hypothesis from a person-centered perspective, highlighting heterogeneous joint patterns of childhood maltreatment and recent stress and their concurrent associations with NSSI. The findings are also broadly consistent with both the self-punishment hypothesis (3) and the Benefits and Barriers Model of NSSI. Specifically, different childhood maltreatment and recent stress profiles showed statistical indirect associations with NSSI through elevated levels of inadequate-self and hated-self (threat-related self-criticism), as well as reduced levels of self-compassion (soothing/safeness-related self-relating). Among these associations, hated-self demonstrated the most prominent and consistent relative indirect association. Practically, the results emphasize the potential value of considering both childhood maltreatment and recent stress exposure as indicators in early mental health screening. This comprehensive approach may facilitate earlier detection of psychological distress. Furthermore, the identification of these self-related processes offers preliminary guidance for targeted prevention and intervention measures aimed at reducing severe self-criticism and promoting supportive self-relating.

These findings should be interpreted in light of several limitations that warrant further investigation. First, the predominance of female participants in the sample may limit the generalizability of the findings to more gender-balanced populations. Future studies should recruit more male participants and examine whether the identified profiles and associations are consistent across genders. Second, the cross-sectional design precludes conclusions regarding causal relationships. Because self-criticism, self-compassion, and NSSI were assessed concurrently, the temporal ordering of the indirect associations could not be established. Thus, these findings should not be interpreted as evidence of causal mediating mechanisms. Subsequent research should employ multiwave longitudinal designs and growth models to examine how childhood maltreatment and changes in recent stress (e.g., across one- or three-year intervals), are prospectively associated with NSSI. Third, one CTQ subscale showed relatively low internal consistency, which may have introduced measurement error, attenuated the observed associations, and reduced the precision of profile classification. Findings involving this subscale should therefore be interpreted cautiously. Fourth, although the CMP profile has potential practical relevance, its small size may reduce the stability of the estimates. Therefore, findings concerning this profile should be interpreted cautiously and validated in larger independent samples. Additionally, all variables were assessed using self-report questionnaires, which may have been influenced by social desirability bias or underreporting of sensitive experiences, particularly for childhood maltreatment and NSSI. Although several measures were implemented to enhance confidentiality and reduce response bias, these influences cannot be completely ruled out and remain an inherent limitation of questionnaire-based research. Furthermore, although the ASLEC has been widely used and validated among Chinese university students and young adults (53–55), it was originally developed for adolescent populations. Therefore, caution is warranted when interpreting recent stress assessed by this measure in young adult samples, and future studies are encouraged to employ stress measures specifically developed or validated for young adults. Finally, although the present study was guided by Gilbert’s evolutionary framework, it focused exclusively on inadequate-self, hated-self, and self-compassion (operationalized as reassured-self). A more nuanced understanding of NSSI requires future work examining the interplay among a broader range of self-related, interpersonal, and emotional processes.

## 5 Conclusion

By simultaneously considering distal adversity (childhood maltreatment) and proximal stress (recent stress) across developmental periods, the present study identified four distinct profiles characterized by childhood maltreatment and recent stress among Chinese young adults. Compared with the LR group, the CMP, SP, and MCMS groups were all significantly associated with a higher probability of NSSI. Furthermore, significant indirect associations through self-criticism—particularly hated-self—and self-compassion were observed between profile membership and NSSI. These findings contribute to a more nuanced understanding of the joint patterns of childhood maltreatment and recent stress and their concurrent associations with NSSI, while providing preliminary guidance for targeted NSSI prevention and intervention efforts among young adults.

## Data Availability

The raw data supporting the conclusions of this article will be made available by the authors, without undue reservation.
