# Supplementary material for: Distinct profiles of childhood maltreatment and recent stress in relation to non-suicidal self-injury: the roles of self-criticism and self-compassion
Source: Front Psychiatry. 2026 Aug 26;17:1898914. doi: 10.3389/fpsyt.2026.1898914 (PMC13553912; doi:10.3389/fpsyt.2026.1898914)
Supplement: Supplementary file 1 [file DataSheet1.docx]

Supplementary Material

# 1 Assessment of Potential Common Method Bias

Because several psychological variables in the present study were assessed using self-report questionnaires, we evaluated the potential influence of common method bias (CMB) using multiple approaches.

## 1.1 Harman’s single-factor test

Harman’s single-factor test was used to examine potential common method bias (Zhou and Long, 2004). The results revealed that the first unrotated factor accounted for 22.41% of the total variance, which is below the critical threshold of 40%. The result indicated that no single factor accounted for the majority of the covariance among the measured variables.

## 1.2 Confirmatory factor analysis comparing alternative measurement models

Confirmatory factor analyses were conducted to evaluate the potential influence of common method bias. The present study included 13 self-reported psychological measures (although NSSI was also assessed through self-report, it was treated as a behavioral indicator reflecting actual behavior rather than a subjective psychological construct, therefore not included in the assessment of common method bias). Among the 13 psychological measures, 10 variables (i.e., childhood trauma and recent stress measures) were only included as indicators for the latent profile analysis and were not incorporated into the subsequent mediation model. In contrast, inadequate-self, hated-self and self-compassion were specified as mediating variables in the theoretical model and were directly examined as predictors of NSSI occurrence through the mediation framework. Because these three constructs represented the psychological pathways tested in the main analyses and were simultaneously assessed using self-report questionnaires, they were selected as the focus of the measurement-based evaluation of common method bias.

We specified two alternative factor models. In the first model, the items corresponding to the three constructs were specified to load onto their respective latent factors according to the original scale structures. In the second model, all 23 items were loaded onto a single common method factor. By comparing the model fit indices of these two models, we evaluated whether the measurement structure was substantially better explained by the theoretically specified factors or by a single common factor. If substantial common method bias were present, the single-factor model would be expected to demonstrate an acceptable level of model fit, indicating that a single underlying factor could adequately account for the covariance among the observed items. Model fit was evaluated using the chi-squared-degree of freedom ratio (*χ2/df*), comparative fit index (CFI), Tucker-Lewis index (TLI), root mean square error of approximation (RMSEA), and standardized root mean residual (SRMR) (Hu and Bentler, 1998).

The results revealed that, the three-factor model demonstrated substantially better fit (*χ²/df* = 38.570, CFI = .901, TLI = .889, RMSEA = .085, SRMR = .064) than the single-factor model (*χ²/df* = 194.596, CFI = .481, TLI = .426, RMSEA = .193, SRMR = .171), with a ΔCFI of .420, suggesting that the covariance among the indicators was unlikely to be explained by a single common method factor.

## 1.3 Unmeasured latent method construct analysis

To further examine the possibility of common method variance, an unmeasured latent method construct (ULMC) model was estimated (Podsakoff et al., 2003). In the model, the three substantive latent factors (inadequate-self, hated-self and self-compassion) were specified based on their respective measurement structures, while an additional latent common method factor was included, with all 23 items loading simultaneously on this method factor.

The results indicated that the inclusion of the latent method factor slightly improved the overall model fit (*χ²/df* = 37.164, CFI = .914, TLI = .893, RMSEA = .083, SRMR = .050). Although the inclusion of the latent method factor resulted in improved global fit indices, the model produced a non-positive definite latent covariance matrix and unstable parameter estimates. Specifically, some substantive latent factors showed estimation problems after introducing the method factor, suggesting that the method factor could not be empirically separated from the substantive constructs. Therefore, the ULMC results were interpreted as a sensitivity analysis rather than a definitive estimate of common method variance.

## 1.4 Consideration of study design characteristics

In addition to statistical assessments, we considered the design characteristics of the present study when evaluating potential common method bias.

First, the predictor variable in the main analyses was latent profile membership derived from multiple psychological indicators rather than a single self-reported variable. Therefore, the predictor represented a data-driven classification based on multiple measures.

Second, the primary outcome variable, NSSI occurrence, was assessed as a behavioral indicator rather than a subjective psychological evaluation. Thus, the predictor and outcome variables were not obtained using the same measurement approach, reducing concerns that the observed associations were solely attributable to shared self-report variance.

Taken together, although common method variance cannot be completely ruled out, the converging evidence from multiple analyses suggests that it is unlikely to substantially account for the observed findings.

# 2 Additional Evaluation of the Four-Profile Solution

## 2.1 Evaluation of Model Estimation Stability: Random Start Specifications

To ensure that the latent profile solutions were not affected by local maxima, all models were estimated using random starts. Specifically, all latent profile analyses were conducted using the same random start specification: 200 sets of random initial stage solutions were generated, and the best 50 solutions were retained for final stage optimization. The best loglikelihood value was replicated across the retained solutions, indicating stable model estimation.

## 2.2 Evaluation of the Small CMP Profile in the Four-Profile Solution

In the four-profile solution, the CMP profile consisted of 116 participants, accounting for only 2.22% of the total sample. To demonstrate that this profile represents a meaningful and interpretable subgroup rather than an artifact of model estimation, we presented the following supplementary evidences.

### 2.2.1 Clinical and theoretical relevance of the CMP profile

Although the CMP group comprised only a small proportion of the sample, this subgroup has important clinical relevance because individuals who experienced childhood maltreatment or neglect represent a particularly vulnerable population with increased vulnerability to psychological problems and self-injurious behaviors. Identifying this subgroup may facilitate targeted screening and early intervention for individuals who may benefit from specialized preventive strategies.

### 2.2.2 Classification information of the profiles

As reported in the main manuscript, although the CMP profile in the four-profile solution consisted of a relatively small number of participants (*n* = 116, 2.22% of the total sample), it demonstrated a high average posterior probability (97.6%) and a low classification error rate (2.4%), indicating high classification certainty despite its relatively small size.

In addition, we further examined the results of the three-profile and five-profile solutions. The results showed that both the three-profile and five-profile solutions also identified a relatively small subgroup (*see* Table S2). Specifically, the smallest profiles included fewer than 150 participants and accounted for less than 3% of the total sample, whereas also demonstrated high posterior classification probabilities.

Regarding symptom characteristics (*see* Figure S2), these small subgroups consistently represented individuals with high levels of childhood trauma across different profile solutions. Therefore, altering the number of extracted profiles alone did not eliminate or substantially change the presence of this small but distinct subgroup, suggesting that this subgroup reflects meaningful heterogeneity within the sample rather than an artifact specific to the four-profile solution.

### 2.2.3 Robustness of the CMP profile

To further examine whether the identification of the CMP profile was driven by any specific indicator, we conducted a series of sensitivity analyses using a leave-one-indicator-out approach. Specifically, the four-profile solution was re-estimated after removing each of the ten indicators individually, resulting in ten alternative four-profile models.

Across all alternative model specifications, a small CMP profile remained consistently identifiable (*see* Table S3). The proportion of participants assigned to this profile ranged from 1.42% to 2.36%, which was comparable to the original four-profile solution (2.22%). The composition of the remaining profiles was also largely comparable across analyses (Table S3). Although removing interpersonal stress resulted in a relatively greater redistribution of participants among the larger profiles, the presence of the CMP profile was retained, supporting the robustness of this subgroup.

## 2.3 Sensitivity analysis using the five-profile solution in logistic regression

To further examine whether the conclusions regarding the association between latent profiles and NSSI occurrence were sensitive to the selection of the four- and five-profile solution, we conducted a sensitivity analysis based on the five-profile model. Following the logistic regression approach used in the main analyses, NSSI occurrence (0 = “no”, 1 = “yes”) was specified as the dependent variable, and dummy-coded latent profile membership derived from the five-profile solution was included as the independent variable. The smallest profile in terms of sample size (i.e., profile 5 in Table S2) was selected as the reference group for dummy coding. Sex, age, only-child status, and financial status were included as covariates, consistent with the main logistic regression analyses.

The Logistic regression results showed that, the smallest profile did not significantly differ from the second smallest profile in predicting NSSI occurrence (OR = 0.818, 95% CI = [0.445, 1.505], *p* = .519), suggesting that the additional profile identified in the five-profile solution did not provide additional explanatory value regarding NSSI occurrence. This finding provides additional evidence supporting the selection of the four-profile solution over the five-profile solution.

# 3 Sensitivity Analysis for Additional Demographic Covariate Adjustment

To ensure that our covariate selection adequately accounted for the potential confounding effects of all measurable demographic variables available in the dataset, we conducted a sensitivity analysis. Specifically, we selected one mediation model presented in the main manuscript, using the LR group as the reference group and the MCMS group as the independent variable. Two additional demographic variables that were not included in the primary models (i.e., type of residence and parental marital status) were additionally incorporated as covariates predicting both the mediator variables and the outcome variable. Because these two variables were categorical, they were dummy-coded into multiple binary variables according to their respective categories.

The results showed that the findings were highly comparable with those from the original model without adjustment for these two additional demographic variables (*see* Table S7). The maximum absolute change in the estimated coefficients was quite small (|*Δβ|* = .003), which remained within an acceptable range. Therefore, these findings suggest that type of residence and parental marital status had limited influence on the mediation model, and the robustness of the original results was supported.

# 4 Supplementary Figures and Tables

## 4.1 Supplementary Tables

**Supplementary Table S1**

**Table S1**. Descriptive statistics of each LPA input indicator

| **Indicator** | **Mean** | ***SD*** | **Skewness** | **Kurtosis** | **Range** |
| --- | --- | --- | --- | --- | --- |
| Emotional abuse | 1.30 | 1.99 | 2.49 | 8.57 | 0 – 18 |
| Physical abuse | 0.32 | 1.07 | 5.33 | 36.87 | 0 – 13 |
| Sexual abuse | 0.27 | 1.03 | 6.07 | 49.19 | 0 – 15 |
| Emotional neglect | 3.56 | 3.49 | 1.23 | 1.49 | 0 – 20 |
| Physical neglect | 2.41 | 2.44 | 1.17 | 1.22 | 0 – 16 |
| Punishment stress | 4.12 | 6.37 | 1.76 | 2.41 | 0 – 35 |
| Academic stress | 6.60 | 4.78 | 0.51 | -0.32 | 0 – 25 |
| Loss stress | 3.85 | 5.14 | 1.50 | 1.34 | 0 – 25 |
| Interpersonal stress | 5.09 | 4.02 | 0.50 | -0.54 | 0 – 20 |
| Adaptation stress | 3.90 | 3.38 | 0.86 | 0.31 | 0 – 19 |

**Supplementary Table S2**

**Table S2**. Profile composition and classification quality of the three-, four and five-class models

| **Group** | ***n*** | **Proportion**  **(%)** | **Average posterior**  **probability** | **Classification**  **error rate** |
| --- | --- | --- | --- | --- |
| **Three-profile classification** | | | | |
| 1 | 4091 | 78.31% | 0.992 | 0.008 |
| 2 | 996 | 19.07% | 0.976 | 0.024 |
| 3 | 137 | 2.62% | 0.983 | 0.017 |
| **Four-profile classification** | | | | |
| LR | 2720 | 52.07% | 0.953 | 0.047 |
| MCMS | 1611 | 30.84% | 0.906 | 0.094 |
| SP | 777 | 14.87% | 0.974 | 0.026 |
| CMP | 116 | 2.22% | 0.976 | 0.024 |
| **Five-profile classification** | | | | |
| 1 | 2643 | 50.42% | 0.959 | 0.050 |
| 2 | 1513 | 28.96% | 0.904 | 0.096 |
| 3 | 744 | 14.24% | 0.971 | 0.029 |
| 4 | 262 | 5.02% | 0.973 | 0.027 |
| 5 | 71 | 1.36% | 0.999 | 0.001 |

**Supplementary Table S3**

**Table S3**. Profile composition of four-profile solutions after omitting individual indicators

| **Variable**  **omitted** | ***n* (%)** | | | |
| --- | --- | --- | --- | --- |
|  | **Profile 1** | **Profile 2** | **Profile 3** | **Profile 4** |
| **EA** | 2696 (51.61%) | 1631 (31.22%) | 778 (14.89%) | 119 (2.28%) |
| **PA** | 2722 (52.11%) | 1617 (30.95%) | 774 (14.82%) | 111 (2.13%) |
| **SA** | 2728 (52.22%) | 1600 (30.63%) | 779 (14.91%) | 117 (2.24%) |
| **EN** | 2713 (51.93%) | 1622 (31.05%) | 776 (14.86%) | 113 (2.16%) |
| **PN** | 2723 (52.13%) | 1610 (30.82%) | 775 (14.84%) | 116 (2.22%) |
| **PS** | 2680 (51.30%) | 1629 (31.18%) | 792 (15.16%) | 123 (2.36%) |
| **ACS** | 3105 (59.44%) | 1240 (23.74%) | 766 (14.66%) | 113 (2.16%) |
| **LS** | 2740 (52.45%) | 1627 (31.15%) | 737 (14.11%) | 120 (2.30%) |
| **IS** | 3988 (76.34%) | 878 (16.81%) | 284 (5.44%) | 74 (1.42%) |
| **ADS** | 2885 (55.23%) | 1440 (27.57%) | 781 (14.95%) | 118 (2.26%) |

*Note*. Profile labels were assigned according to the descending order of class size (i.e., Profile 1 represents the largest subgroup and Profile 4 represents the smallest subgroup) within each sensitivity analysis. Profile labels do not indicate any ranking or clinical severity. Values are presented as *n* (%). EA: emotional abuse; PA: physical abuse; SA: sexual abuse; EN: emotional neglect; PN: physical neglect; PS: punishment stress; ACS: academic stress; LS: loss stress; IS: interpersonal stress; ADS: adaptation stress

**Supplementary Table S4**

**Table S4**. Means (standard deviations) of childhood trauma and recent stress across different profiles and results of post hoc comparisons

|  | **EA** | **PA** | **SA** | **EN** | **PN** | **PS** | **ACS** | **LS** | **IS** | **ADS** |
| --- | --- | --- | --- | --- | --- | --- | --- | --- | --- | --- |
| **CMP group (A)** | 6.267 | 5.853 | 2.379 | 9.560 | 5.750 | 6.819 | 9.431 | 5.431 | 7.802 | 6.224 |
|  | (3.475) | (2.323) | (3.139) | (4.239) | (3.635) | (6.703) | (5.031) | (4.824) | (4.133) | (3.870) |
| **SP group (B)** | 1.732 | 0.310 | 0.468 | 5.327 | 3.445 | 16.456 | 12.360 | 13.708 | 9.996 | 8.570 |
|  | (2.040) | (0.743) | (1.393) | (3.617) | (2.699) | (5.496) | (3.324) | (3.877) | (2.793) | (2.758) |
| **MCMS group (C)** | 1.880 | 0.295 | 0.280 | 4.315 | 2.942 | 3.953 | 9.220 | 3.838 | 7.406 | 5.084 |
|  | (2.230) | (0.709) | (0.894) | (3.583) | (2.382) | (3.935) | (3.443) | (3.354) | (2.821) | (2.610) |
| **LR group (D)** | 0.624 | 0.096 | 0.126 | 2.634 | 1.651 | 0.585 | 3.289 | 0.980 | 2.207 | 1.767 |
|  | (1.098) | (0.418) | (0.621) | (2.903) | (1.984) | (1.391) | (2.805) | (1.632) | (2.174) | (1.815) |
| **χ^2^** | 717.178 | 878.423 | 306.953 | 326.640 | 466.392 | 2339.369 | 2615.750 | 2122.690 | 2861.924 | 2157.318 |
| ***p*** | .000 | .000 | .000 | .000 | .000 | .000 | .000 | .000 | .000 | .000 |
| **Pairwise Comparisons** | A>B=C>D | A>B=C>D | A>B>C>D | A>B=C>D | A>B>C>D | B>A>C>D | B>A=C>D | B>A=C>D | B>A=C>D | B>A>C>D |

*Note*. The significance level for post hoc comparisons was set at .05 and adjusted using the Bonferroni correction. CMP group: childhood maltreatment predominant group (A); SP group: stress predominant group (B); MCMS group: medium CM & stress group (C); LR group: low risk group (D). EA: emotional abuse; PA: physical abuse; SA: sexual abuse; EN: emotional neglect; PN: physical neglect; PS: punishment stress; ACS: academic stress; LS: loss stress; IS: interpersonal stress; ADS: adaptation stress

**Supplementary Table S5**

**Table S5.** The logistic regression of latent profiles and NSSI occurrence

| **Variables** | **Model results** | | |
| --- | --- | --- | --- |
|  | ***β*** | **OR (95% CI)** | ***p*** |
| Sex | 0.382 | 1.466 (1.269,1.692) | .000 |
| Age | −0.107 | 0.898 (0.877, 0.920) | .000 |
| Only child | 0.155 | 1.168 (1.012, 1.347) | .034 |
| Financial status | −0.241 | 0.786 (0.663, 0.930) | .005 |
| **Reference category: Low risk group** | | | |
| Intercept | 0.453 |  | .085 |
| Medium CM & stress group | 1.054 | 2.869 (2.457, 3.350) | .000 |
| Stress predominant group | 1.265 | 3.542 (2.924, 4.291) | .000 |
| CM predominant group | 1.833 | 6.253 (3.994, 9.790) | .000 |
| **Reference category: Medium childhood maltreatment & stress group** | | | |
| Intercept | 1.507 |  | .000 |
| Low risk group | −1.054 | 0.349 (0.299, 0.407) | .000 |
| Stress predominant group | 0.211 | 1.235 (1.021, 1.494) | .030 |
| CM predominant group | 0.779 | 2.180 (1.393, 3.410) | .000 |
| **Reference category: Stress predominant group** | | | |
| Intercept | 1.718 |  | .000 |
| Low risk group | −1.265 | 0.282 (0.233, 0.342) | .000 |
| Medium CM & stress group | −0.211 | 0.810 (0.670, 0.980) | .030 |
| CM predominant group | 0.568 | 1.765 (1.115, 2.796) | .015 |
| **Reference category: Childhood maltreatment predominant group** | | | |
| Intercept | 2.287 |  | .000 |
| Low risk group | −1.833 | 0.160 (0.102, 0.250) | .000 |
| Medium CM & stress group | −0.779 | 0.459 (0.293, 0.718) | .000 |
| Stress predominant group | −0.568 | 0.566 (0.358, 0.897) | .015 |

*Note.* Using a different reference category did not change the regression of the control variables, so the coefficients remained unchanged. *OR*: odd ratio. Sex, age, only-child and financial status were included as covariate, coded as follows: male = 0, female = 1; only child = 1, not only child = 0; financial difficulty = 1, no financial difficulty = 0.

**Supplementary Table S6**

**Table S6.** Detailed coefficients for all six mediating models

| **Path** | **CM predominant group** | | | **Stress predominant group** | | | **Medium CM & stress group** | | |
| --- | --- | --- | --- | --- | --- | --- | --- | --- | --- |
|  | ***β*** | ***p*** | **95%CI** | ***β*** | ***p*** | **95%CI** | ***β*** | ***p*** | **95%CI** |
| Inadequate-self → NSSI | 0.076 | .001 | [0.034, 0.122] | 0.076 | .001 | [0.034, 0.122] | 0.076 | .001 | [0.034, 0.122] |
| Hated-self → NSSI | 0.201 | .000 | [0.161, 0.238] | 0.201 | .000 | [0.161, 0.238] | 0.201 | .000 | [0.161, 0.238] |
| Self-compassion → NSSI | −0.097 | .000 | [−0.133, −0.058] | −0.097 | .000 | [−0.133, −0.058] | −0.097 | .000 | [−0.133, −0.058] |
| **Reference category: Low risk group** | | | | | | | | | |
| Profile → NSSI | 0.085 | .000 | [0.051,0.122] | 0.157 | .000 | [0.119, 0.193] | 0.175 | .000 | [0.136, 0.214] |
| Profile → Inadequate-self | 0.119 | .000 | [0.084, 0.153] | 0.293 | .000 | [0.268, 0.321] | 0.354 | .000 | [0.329, 0.381] |
| Profile → Hated-self | 0.137 | .000 | [0.100, 0.173] | 0.256 | .000 | [0.224, 0.289] | 0.227 | .000 | [0.195, 0.255] |
| Profile → Self-compassion | −0.149 | .000 | [−0.190, −0.109] | −0.132 | .000 | [−0.161, −0.101] | −0.161 | .000 | [−0.191, −0.132] |
| Indirect effects: inadequate-self | 0.009 | .003 | [0.004, 0.016] | 0.022 | .000 | [0.010, 0.036] | 0.027 | .001 | [0.012, 0.043] |
| Indirect effects: hated-self | 0.027 | .000 | [0.019, 0.037] | 0.051 | .000 | [0.040, 0.065] | 0.046 | .000 | [0.035, 0.056] |
| Indirect effects: self-compassion | 0.014 | .000 | [0.008, 0.022] | 0.013 | .000 | [0.008, 0.019] | 0.015 | .000 | [0.009, 0.023] |
| Total indirect effects | 0.051 | .000 | [0.038, 0.062] | 0.087 | .000 | [0.072, 0.103] | 0.088 | .000 | [0.072, 0.105] |
| **Reference category: Medium CM & stress group** | | | | | | | | | |
| Profile → NSSI | 0.034 | .056 | [0.000, 0.070] | 0.023 | .226 | [−0.014, 0.060] |  |  |  |
| Profile → Inadequate-self | 0.016 | .334 | [−0.018, 0.047] | 0.022 | .121 | [−0.005, 0.050] |  |  |  |
| Profile → Hated-self | 0.070 | .000 | [0.035, 0.107] | 0.082 | .000 | [0.048, 0.117] |  |  |  |
| Profile → Self-compassion | −0.102 | .000 | [−0.141, −0.064] | −0.009 | .574 | [−0.038, 0.022] |  |  |  |
| Indirect effects: inadequate-self | 0.001 | .380 | [−0.001, 0.004] | 0.002 | .172 | [−0.000, 0.005] |  |  |  |
| Indirect effects: hated-self | 0.014 | .000 | [0.007, 0.022] | 0.017 | .000 | [0.009, 0.025] |  |  |  |
| Indirect effects: self-compassion | 0.010 | .001 | [0.005, 0.016] | 0.001 | .548 | [−0.002, 0.004] |  |  |  |
| Total indirect effects | 0.025 | .000 | [0.014, 0.036] | 0.019 | .000 | [0.009, 0.030] |  |  |  |
| **Reference category: Stress predominant group** | | | | | | | | | |
| Profile → NSSI | 0.025 | .164 | [−0.010, 0.061] |  |  |  |  |  |  |
| Profile → Inadequate-self | 0.007 | .657 | [−0.027, 0.039] |  |  |  |  |  |  |
| Profile → Hated-self | 0.039 | .039 | [0.003, 0.076] |  |  |  |  |  |  |
| Profile → Self-compassion | −0.099 | .000 | [−0.138, −0.059] |  |  |  |  |  |  |
| Indirect effects: inadequate-self | 0.001 | .676 | [−0.002, 0.004] |  |  |  |  |  |  |
| Indirect effects: hated-self | 0.008 | .043 | [0.001, 0.016] |  |  |  |  |  |  |
| Indirect effects: self-compassion | 0.010 | .001 | [0.005, 0.016] |  |  |  |  |  |  |
| Total indirect effects | 0.018 | .002 | [0.007, 0.029] |  |  |  |  |  |  |

*Note.* In structural equation modeling, the distinction in the reference category is reflected solely in the coding method of the independent variables. Therefore, in different models, the latter part of the mediation effect, i.e., the regression coefficients for inadequate-self, hated-self, and self-compassion in relation to the occurrence of NSSI, remain unchanged.

**Table S7.** Sensitivity analysis of mediation models with additional demographic covariate adjustment

| **Path** | **Original model** | | | **Sensitive model** | | |
| --- | --- | --- | --- | --- | --- | --- |
|  | ***β*** | ***p*** | **95%CI** | ***β*** | ***p*** | **95%CI** |
| Inadequate-self → NSSI | 0.076 | 0.001 | [0.034, 0.122] | 0.079 | 0.000 | [0.037,0.125] |
| Hated-self → NSSI | 0.201 | 0.000 | [0.161, 0.238] | 0.201 | 0.000 | [0.161,0.238] |
| Self-compassion → NSSI | −0.097 | 0.000 | [−0.133, −0.058] | −0.097 | 0.000 | [−0.133, −0.058] |
| Profile → NSSI | 0.175 | 0.000 | [0.136, 0.214] | 0.176 | 0.000 | [0.138,0.215] |
| Profile → Inadequate-self | 0.354 | 0.000 | [0.329, 0.381] | 0.353 | 0.000 | [0.327,0.379] |
| Profile → Hated-self | 0.227 | 0.000 | [0.195, 0.255] | 0.226 | 0.000 | [0.196,0.254] |
| Profile → Self-compassion | −0.161 | 0.000 | [−0.191, −0.132] | −0.160 | 0.000 | [−0.191, −0.131] |

*Note.* Both models used the LR group as the reference group, and the MCMS group as the independent variable. The left panel presents the original model without adjustment for type of residence and parental marital status (identical to the results presented in Table S6), whereas the right panel presents the model additionally adjusted for these two demographic covariates.

## 4.2 Supplementary Figures

**Supplementary Figure S1**

**Figure S1**. Correlation analysis of the study variables

*Note*. ****p* < 0.001, ***p* < 0.01, **p* < 0.05

**Supplementary Figure S2**

**Figure S2**. Latent Profiles results of Childhood maltreatment and recent stress 3 class and 5 class solution

*Note*. In latent profile analysis, each dimension’s score was converted to a standardized score

# Reference

Hu, L.-T. & Bentler, P. M. 1998. Fit indices in covariance structure modeling : Sensitivity to underparameterized model misspecification. *Psychological Methods,* 3**,** 424-453.

Podsakoff, P., Mackenzie, S., Lee, J.-Y. & Podsakoff, N. 2003. Common Method Biases in Behavioral Research: A Critical Review of the Literature and Recommended Remedies. *Journal of Applied Psychology,* 88**,** 879-903.

Zhou, H. & Long, L. 2004. Statistical tests and control methods for common method bias. *Advances in Psychological Science,* 12**,** 942 - 942.
